# Structure-Guided Mechanisms Behind the Metabolism of 2,4,6-Trinitrotoluene by Glutathione Transferases U25 and U24 That Lead to Alternate Product Distribution

**DOI:** 10.3389/fpls.2018.01846

**Published:** 2018-12-12

**Authors:** Kyriakos Tzafestas, Laziana Ahmad, M. Paulina Dani, Gideon Grogan, Elizabeth L. Rylott, Neil C. Bruce

**Affiliations:** ^1^Centre for Novel Agricultural Products, Department of Biology, University of York, York, United Kingdom; ^2^York Structural Biology Laboratory, Department of Chemistry, University of York, York, United Kingdom

**Keywords:** 2,4,6-trinitrotoluene, TNT, Arabidopsis, glutathione transferase, GST, detoxification, xenobiotic

## Abstract

The explosive xenobiotic 2,4,6-trinitrotoluene (TNT) is a major worldwide environmental pollutant and its persistence in the environment presents health and environmental concerns. The chemical structure of TNT dictates that biological detoxification pathways follow predominantly reductive transformation of the nitro groups, and as a result, TNT is notoriously recalcitrant to mineralization in the environment. Plant-based technologies to remediate this toxic pollutant rely on a solid understanding of the biochemical detoxification pathways involved. Toward this, two Arabidopsis Tau class glutathione transferases, GSTU24 and GSTU25, have been identified that catalyze the formation of three TNT-glutathionylated conjugates. These two GSTs share 79% identity yet only GSTU25 catalyzes the substitution of a nitro group for sulfur to form 2-glutathionyl-4,6-dinitrotoluene. The production of this compound is of interest because substitution of a nitro group could lead to destabilization of the aromatic ring, enabling subsequent biodegradation. To identify target amino acids within GSTU25 that might be involved in the formation of 2-glutathionyl-4,6-dinitrotoluene, the structure for GSTU25 was determined, in complex with oxidized glutathione, and used to inform site-directed mutagenesis studies. Replacement of five amino acids in GSTU24 established a conjugate profile and activity similar to that found in GSTU25. These findings contribute to the development of plant-based remediation strategies for the detoxification of TNT in the environment.

## Introduction

The continual use of explosives, along with production and decommissioning is progressively contaminating military sites worldwide (Amaral et al., 2009; Zheng et al., 2009). The total area of operational ranges in the United States contaminated with munitions constituents is estimated to be more than 10 million hectares (United States General Accounting Office, 2004). Pollution in European countries, from former WWII manufacturing and disposal sites is also widespread (Spain et al., 2000). The most broadly used explosive, 2,4,6-trinitrotoluene (TNT) is associated with extensive soil and water contamination (Lewis et al., 2004). Contaminated training ranges have hotspots of TNT that can reach concentrations of up to 87000 mg kg^-1^ soil (Talmage et al., 1999), with 100–1000 mg kg^-1^, or lower for surface soils in artillery training ranges and 1–36 mg kg^-1^ for hand grenade ranges (Jenkins et al., 2006; Clark and Boopathy, 2007).

Nitro-substituted organic compounds, such as TNT, pose a specific challenge to plant and bacterial degradation. The electron withdrawing nitro groups on the TNT molecule provide stability to the aromatic ring through resonance, rendering the ring particularly resistant to oxidative attack and subsequent ring cleavage (Qasim et al., 2009). Thus TNT is particularly recalcitrant to biodegradation and persists in the environment (Rylott et al., 2011).

**FIGURE 1:**
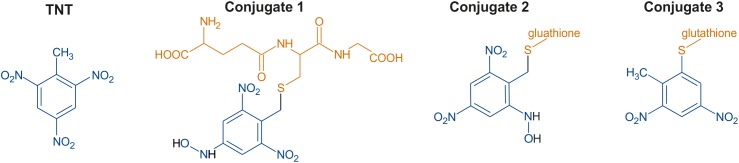
Chemical structures of 2,4,6-trinitrotoluene (TNT) and the three glutathione-TNT conjugates, as determined by Gunning et al. (2014).

In a previous study, two *Arabidopsis thaliana* (Arabidopsis) glutathione transferases, GSTU24 and GSTU25, were shown to conjugate TNT to glutathione (GSH) producing three distinct TNT-GSH conjugates, shown in Figure 1 (Gunning et al., 2014). For two of the compounds, GSH conjugation occurred through the methyl group of TNT; however, the third conjugate (conjugate 3) resulted from the nucleophilic substitution of a nitro group to form 2-glutathionyl-4,6-dinitrotoluene (GDNT). Replacement of the nitro group with sulfur could destabilize the aromatic ring. Fungi and bacteria with the ability to mineralize dinitrotoluenes exist (Valli et al., 1992; Nishino et al., 2000; Johnson et al., 2002) and enzymatic pathways for DNT biodegradation have been characterized (Nishino et al., 2000; Johnson et al., 2002). Thus, production of GDNT could present an opportunity for cleavage and subsequent biodegradation of this toxic environmental pollutant.

Plant GSTs are a superfamily of enzymes: In Arabidopsis, there are 54 GSTs subdivided into seven classes. While many GSTs are able to conjugate GSH to a wide range of xenobiotic substrates, they are also involved in catalyzing ascorbate recycling and various metabolic reactions, with some GSTs also exhibiting glutathione peroxidase (GPOX) activity (Dixon and Edwards, 2010), and non-enzymatic ligand binding properties (Smith et al., 2003; Dixon et al., 2011). The Tau class, to which GSTU24 and GSTU25 belong, can be subdivided into three distinct clades. Many of the GSTs within the clade GSTU19 to GSTU28 are implicated in the detoxification of xenobiotics such as herbicides and safeners (Edwards et al., 2005; Labrou et al., 2015). Expression of both GSTU24 and GSTU25 is induced by TNT, with GSTU25 also exhibiting relatively high GPOX activity (Dixon and Edwards, 2009) and activity toward the model substrate 1-chloro-2,4-dinitrobenzene (CDNB; Mezzari et al., 2005; Gandia-Herrero et al., 2008). To date, Tau class GSTs are unique in their ability to bind glutathione-conjugated fatty acid derivatives (Mezzari et al., 2005; Dixon and Edwards, 2009), with GSTU25 known to selectively bind hydroxylated fatty acids. Yet, despite the mounting knowledge on these enzymes, the endogenous roles for GSTU24 and GSTU25, and the vast majority of plant GSTs in general, remains elusive.

The structures of several Tau class plant GSTs have been solved: The wheat (*Triticum aestivum*), *Ta*GSTU4-4 structure was determined in complex with *S*-hexylglutathione (Thom et al., 2002) and rice (*Oryza sativa*) *Os*GSTU1 (Protein Data Bank code 1OYJ), while two soybean (*Glycine max*) GSTs have been determined; *Gm*GST-U4-4 in complex with *S*-(*p*-nitrobenzyl)-glutathione (Axarli et al., 2009) and *Gm*GSTU10-10 (Skopelitou et al., 2015). Although there is high protein sequence variability between these GSTs, the structures are remarkably conserved (Dixon and Edwards, 2010; Skopelitou et al., 2015). Existing as soluble homo or heterodimers, each 23–30 kDa subunit is 200–300 amino acids in length. Within each subunit is a kinetically independent active site containing G and H sites. The G site, which is relatively well conserved, is formed from the N-terminal domain which exhibits α/β topology, and binds GSH and, less commonly, other closely related peptides. The H-site exists within an α-helical structure in the C-terminal domain but is less well conserved than the G site and, as a result, GSTs have wide substrate specificity.

Only 1.3 kb apart on chromosome I, GSTU24 and GSTU25 share 79% protein identity, indicative of a relatively recent gene duplication event. In this study, we report the structure of GSTU25. We then use this structure, in combination with alignment from other Tau-class plant GSTS whose structures have previously been solved (Axarli et al., 2009, 2016), to predict the key amino acids in the active site of GSTU25 that are associated with the specificity of the conjugation reactions of TNT with GSH.

## Materials and Methods

### Expression and Protein Purification for Crystallization

The GSTU24 and GSTU25 from *A. thaliana* (Arabidopsis) ecotype Col0, and mutants, were cloned from pET-YSBLIC3C (described below) into pET22a to remove the his-tag, then transformed into *Escherichia coli* Tuner (DE3) cells (Novagen) that also contained the pRARE plasmid from Rosetta (Novagen). Transformants were grown on agar plates of Luria Bertani medium containing kanamycin (100 μg mL^-1^) and 50 μg mL^-1^ chloramphenicol (50 μg mL^-1^) (LB+KC). A single colony of a plate grown overnight was used to inoculate a 5 mL starter culture of LB+KC medium, which was grown overnight at 37°C, 180 rpm. The starter culture was then used to inoculate 400 mL LB+KC medium which was incubated at 37°C with shaking until an OD_600_ of 0.5–0.8 was reached. At this point expression of the GST was induced by the addition of isopropyl β-D-1-thiogalactopyranoside (IPTG, final concentration of 1 mM) and culture incubated at 20°C, 180 rpm. After approximately 18 h growth, cells were harvested by centrifugation at 5000 *g* for 15 min then resuspended in 20 mM Tris/HCl buffer pH 7.5. Cells were disrupted by ultrasonication, centrifuged at 15,000 *g* for 30 min then the supernatant loaded onto a 10 mL GSH Sepharose 4B (GE healthcare). Column fractions were analyzed by SDS-PAGE and the fraction containing purified proteins were pooled and concentrated using a 10 kDa cut-off Centricon^®^ filter membrane. Concentrated protein was loaded onto an S75 Superdex^TM^ gel filtration column and fractions containing pure protein, as determined by SDS-PAGE, were pooled and stored at -20°C.

### Protein Crystallization

Commercially available crystallization screens in 96-well plate sitting drop format were pre-incubated with 2 mM GSH and 2 mM TNT in 54 μL of reservoir solution in reservoir well. Pure *At*GSTU25 was then subjected to crystallization trials using a Mosquito^®^ ROBOT (TTP LabTech) in which each drop contained 150 nL protein and 150 nL precipitant reservoir solution. Initial crystals observed for the complex of *At*GSTU25 mixture were obtained in solutions containing 0.2 M ammonium acetate, 0.1 M bis-tris propane pH 5.5 and 25% (w/v) PEG 3350. Larger crystals for diffraction analysis were obtained using the hanging-drop vapor diffusion method in 24-well plate Linbro dishes with 2 μL drops of a ratio of mother liquor to protein solution. The best crystals of the complex of GSTU25 with oxidized glutathione were obtained in crystal drops containing 0.2 M ammonium acetate, 0.1 M bis-tris propane pH 5.5 and 23% (w/v) PEG 3350. Prior to analysis on in-house X-ray equipment, the crystals were washed with the reservoir solution containing 20% (v/v) ethylene glycol as the cryoprotectant, followed by flash-cooling in the liquid nitrogen. Crystals were tested for diffraction using a Rigaku Micromax-007HF fitted with Osmic multilayer optics and a MARRESEARCH MAR345 imaging plate detector. Those crystals that diffracted to a resolution of equal to, or better than, 3 Å were retained for dataset collection at the synchrotron.

### Data Collection, Structure Solution, Model Building, and Refinement

The complete dataset described in this report was collected at the Diamond Light Source, Didcot, United Kingdom on beamline I02. The data were processed and integrated using XDS (Kabsch, 2010) and scaled using SCALA (Evans, 2006) included in the Xia2 processing system (Winter, 2010). Data collection statistics are provided in Table 1. All crystals of U25 were obtained in space group *P*2_1_2_1_2_1_, with four molecules in the asymmetric unit, constituting two dimers. The structure of *At*GSTU25 was solved using MOLREP (Vagin and Teplyakov, 1997), with a monomer of the structure of the Tau class glutathione transferase from *G. max* (PDB 4TOP; 65% sequence identity) as a model. The solvent content in crystals was 51%. Structures were built and refined using iterative cycles using Coot (Emsley and Cowtan, 2004) and REFMAC (Murshudov et al., 1997), employing local NCS restraints in the refinement cycles. After building and refinement of the protein and water molecules, clear residual density was observed in the omit maps at the GSH binding site. This could be clearly modeled as glutathione disulfide (GSSG). The final structure exhibited *R*_cryst_ and *R*_free_ values of 20.5 and 21.7%, respectively. All structures were validated and checked using PDB validation software upon deposition. Refinement statistics for all structures are presented in Table 1. The Ramachandran plot for *At*GSTU25-GSSG showed 98.4% of residues to be situated in the most favored regions, 1.1% in additional allowed and 0.5% residues in outlier regions.

**Table 1:** Data collection and refinement statistics for GSTU25-GSSG complex.

Beamline	Diamond I02
Wavelength (Å)	0.97949
Resolution (Å)	48.54–1.95 (1.99–1.95)
Space group	*P*2_1_2_1_2_1_
Unit cell (Å)	*a* = 87.83; *b* = 107.67; *c* = 108.75’ α = β = γ = 90°
No. of molecules in the asymmetric unit	4
Unique reflections	75638 (4446)
Completeness (%)	99.8 (100.0)
*R*_merge_ (%)	0.07 (0.54)
*R*_p.i.m._	0.05 (0.36)
Multiplicity	6.4 (6.2)
<*I*/*σ(I)*>	12.7 (3.0)
Overall *B* factor from Wilson plot (Å^2^)	25
*R_cryst_/R_free_* (%)	20.5/21.7
r.m.s.d 1–2 bonds (Å)	0.02
r.m.s.d 1–3 angles (°)	1.94
Avge main chain B (Å^2^)	31
Avge side chain B (Å^2^)	35
Avge water B (Å^2^)	42
Avge ligand B (Å^2^)	40


*Numbers in brackets refer to data for highest resolution shells.*

### Generation of the GST Mutants

A QuickChange II Site-Directed Mutagenesis Kit (Agilent Technologies) was used to generate the mutants, using the primers listed in Table 2. Wild-type and mutated GSTU24 and GSTU25 were cloned into pET-YSBLIC3C, used to transform *E. coli* (BL21) cells, and expressed and purified as described previously (Gunning et al., 2014).

**Table 2:** Primers used for the site-directed mutagenesis of GSTU24 and GSTU25.

GSTU24		
**Mutation**	**Primer set**	**Primer sequence (5′–>3′)**

Ala12Pro	U24-A12P-F	GGCAGATGAGGTGATTCTTCTGGATTTCTGGCCGAGTATGTTTGGG
	U24-A12P-R	GCCAGAGCAATTCTTGTCCTCATCCCAAACATACTCGGCCAGAAATC
Asn107Tyr	U24-N107Y-F	CTGGGCCGACTTCATCGACAAAAAGGTGTATGTTACGGCGAG
	U24-N107Y-R	GACCGCCCAAATCCTTCTCGCCGTAACATACACCTTTTTGTCG
Ala115Gly	U24-A115G-F	GGTGAATGTTACGGCGAGAAGGATTTGGGGGGTCAAAGG
	U24-A115G-R	GCTGCTTCTTGCTCCTCACCTTTGACCCCCCAAATCC
Ala115Gly^∗^	U24-A115Gb-F	GGTGTATGTTACGGCGAGAAGGATTTGGGGGGTCAAAGG
	U24-A115Gb-R	Same as U24-A115G-R
Ile208Val	U24-I208V-F	GCCCTGCCTGAGTCAGAGAAGGTCATTACATTCGTTTCCGAACG
	U24-I208V-R	CTCCAACCCAAGTTTCTTCCTACGTTCGGAAACGAATGTAATG
Arg211Leu	U24-R211L-F	GGTCATTACATTCATTTCCGAACTTAGGAAGAAACTTGGGTTGG
	U24-R211L-R	CTCCAACCCAAGTTTCTTCCTAAGTTCGGAAATGAATGTAATGACC
Arg211Leu^∗^	U24-R211Lb-F	GGTCATTACATTCGTTTCCGAACTTAGGAAGAAACTTGGGTTGG
	U24-R211Lb-R	CTCCAACCCAAGTTTCTTCCTAAGTTCGGAAACGAATGTAATGACC
Pro12Ala	U25-P12A-F	GGCAGACGAGGTGATTCTTCTTGATTTCTGGGCGAGCATG
	U25-P12A-R	GCAATCCTCGTCCTCATTCCAAACATGCTCGCCCAGAAATC
Tyr107Asn	U25-Y107N-F	GGCCAAATTTTGGGGAGATTTCATTGATAAGAAGGTGAATGCTTCAGC
	U25-Y107N-R	GCTCCCCAAATCAACCTCGCTGAAGCATTCACCTTCTTATC
Gly115Ala	U25-G115A-F	GGTGTATGCTTCAGCGAGGTTGATTTGGGCAGCTAAAGGC
	U25-G115A-R	CGCCTCATGCTCTTCGCCTTTAGCTGCCCAAATCAACCT
Gly115Ala^∗^	U25-G115Ab-F	GGTGAATGCTTCAGCGAGGTTGATTTGGGCAGCTAAAGGC
	U25-G115Ab-R	Same as U25-G115A-R
Val209Ile	U25-V209I-F	GTCTCTTCCTGATTCGGAGAAGATCATTAAGTTCATTCCTGAGC
	U25-V209I-R	CCCAAGTTTTTTCCTTAGCTCAGGAATGAACTTAATGATCTTCTCCG
Leu212Arg	U25-L212R-F	CGGAGAAGATCATTAAGTTCGTTCCTGAGCGAAGGAAAAAAC
	U25-L212R-R	CTATTCGATTTCGATCCCAAGTTTTTTCCTTCGCTCAGGAACG
Leu212Arg^∗^	U25-L212Rb-F	CGGAGAAGATCATTAAGTTCATTCCTGAGCGAAGGAAAAAAC
	U25-L212Rb-R	CTATTCGATTTCGATCCCAAGTTTTTTCCTTCGCTCAGGAATG


*The asterisks (^∗^) mark primer sets that were designed for the generation of sequential mutations and carry in their sequence the previous mutation, e.g., the Ala115Gly^∗^ primer set is designed to insert the Ala115Gly mutation into a sequence that already has the Asn107Tyr mutation.*

### GST Assays Using CDNB

Conjugating activity of the purified proteins, and crude extracts from rosette leaves, was assessed using CDNB as described previously (Colville and Smirnoff, 2008). Briefly, the reaction, at 25°C, comprised 100 mM potassium phosphate buffer pH 6.5, 5 mM GSH and a range of CDNB concentrations, and was initiated by addition of 5 μg of purified enzyme to a final volume of 1 mL. Increase in absorbance at A_340_ was measured spectrophotometrically. The Michaelis–Menten plots and kinetic calculations (K_m_ and V_max_) were performed using SigmaPlot 14 software. Statistical analysis was performed using Statistical Package for Social Sciences (SPSS) software (version 25, SPSS, Inc., Chicago, IL, United States). Results were analyzed using the analysis of variance (ANOVA) for continuous variables. *P*-values <0.05 were considered to be statistically significant.

### GST Assays Using TNT

Reactions, carried out at 20°C, contained 100 mM potassium phosphate buffer pH 7.0, 300 μg of purified enzyme and 5 mM GSH and was initiated by addition of TNT to a final volume of 250 μL. Reactions were stopped by the addition of trichloroacetic acid, to a final concentration of 10% (v/v), and samples analyzed by HPLC.

### Measurement of TNT and Products

The TNT and conjugates were analyzed by HPLC using a Waters HPLC system (Waters 2695 separator and Waters Photodiode array detector) with Waters X-Bridge C18 column (300 mm × 4.5 mm, 5 μM). The mobile phases for the gradient conditions were: mobile phase A, acetonitrile; mobile phase B, 50 mM NaH_2_PO_4_, pH 2.7, with 85% (v/v) phosphoric acid. The gradient ran: 0 min 0% A 100% B, 6 min 0% A 100% B, 11 min 50% A 50% B, 25 min 100% A 0% B, 30 min 0% A 100% B, runtime 30 min. Integration was performed at 250 nm with Empower Pro Software.

### Nitrite Measurement

Nitrite production was assayed according to the method of French et al. (1998) with modifications as described in Gunning et al. (2014).

### Probing the Mutants With ANS

The ANS binding assay, based on the protocol by Yang et al. (2009), was used to determine conformational changes. The assay was performed in a 1 mL cuvette with 100 μl of 2 mM ANS, 50 μg of enzyme and 100 mM potassium phosphate buffer pH 6.5. The fluorescence emission was monitored using a FluoroMax^®^-4 Spectrofluorometer (Horiba Scientific).

### Accession Numbers

*At*GSTU24; TAIR accession number At1g17180, *At*GSTU25; TAIR acc. no. At1g17170. *At*GSTU25-GSSG coordinates; Protein Databank (PDB) acc. no. 5g5a. *Gm*GSTU4-4; PDB acc. no. 2VO4, Sh14; PDB acc. no. 5AGY, *Pc*Ure2pA; PDB acc. no. 4F0B, *Ec*YghU; PDB acc. no. 3C8E, *Ec*YfcG; PDB acc. no. 3GX0, *Co*GRX2; PDB acc. no. 4TR0.

## Results

### Structure of GSTU25

**FIGURE 2:**
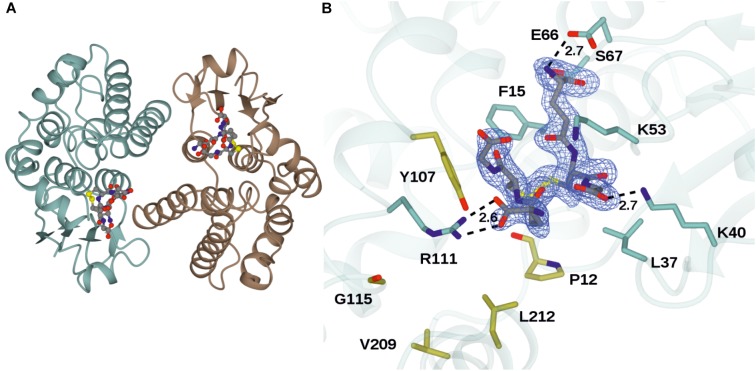
Structures of GSTU25 and target residues. **(A)** Structure of the GSTU25 dimer, with monomers in blue and brown. Oxidized glutathione can be observed in each of the monomer active sites, in stick format. **(B)** Active site of the GSTU25 monomer showing binding of GSSG. The electron density corresponds to the F_o_-F_c_ omit map contoured at a level of 3σ, and is that which was obtained prior to refinement of the ligand atoms, which have been added from the refined ligand complex for clarity. Side chains of residues conserved between U24 and U25 are shown with side-chain carbon atoms in Blue; Side-chains of residue positions chosen for mutation are shown with side-chain carbon atoms in gold.

The structure of GSTU25 was solved using molecular replacement at a resolution of 1.99 Å with *Gm*GSTU4-4 as template (Axarli et al., 2009). Analysis of the protein structure using the DALI server (Holm and Rosenstrom, 2010) revealed that the monomer was more similar to the structure of a Tau class GST mutant from *G. max*, called Sh14 (Axarli et al., 2016). Both structures were 68% identical, with a RMS value of 1.2 Å over 219 residues. Each monomer of GSTU25 has four β-strands and nine α-helices adopting the canonical GST fold. The first 77 residues at the N-terminus fold into a thioredoxin-like domain followed by an α-helical domain at the C-terminus from position 89 to 216, with the two domains connected together by a short linker. Although the crystals were incubated with TNT and GSH, binding of TNT was not detected. Instead, multiple rounds of structure refinement cycles using the REFMAC5 program (Murshudov et al., 2011) revealed two GSH molecules covalently linked by a disulfide bond, showing the structure of GSTU25 in complex with glutathione disulfide (GSSG) (Figure 2A). At the binding site, the GSSG subunits: GSH-1 and GSH-2, were located in a binding pocket surrounded by polar, non-polar and charged amino acids (Figure 2B). This pocket was similar to the active site identified for most GSTs, where the hydroxyl group of S13 and Y107 has been shown to contribute to the ionization of the GSH sulfhydryl group (Brock et al., 2013). Similar locations were observed for the same S and Y residues of *Gm*GSTU4-4 in complex with *S*-(*p*-nitrobenzyl)-glutathione (Axarli et al., 2009). The S residue was found to stabilize the thiolate anion of GSH and enhance its nucleophilicity, while the Y residue was important in regulating catalytic function. The GSTU25-GSSG structure also revealed that the terminal carboxylate group of the GSH-1 γ-glutamyl moiety formed an interaction at 2.6 Å with the nitrogen atom of the guanidinium group of R111, and that the glycine moiety of GSH-1 protruded toward the GSTU25 α4 chain. The GSH-2 molecule, at the carboxylate terminal of the glycine moiety, formed an interaction with the oxygen atom of K40 at a distance of 2.7 Å with the γ-glutamyl moiety located in between the helices α1 and α3.

**FIGURE 3:**
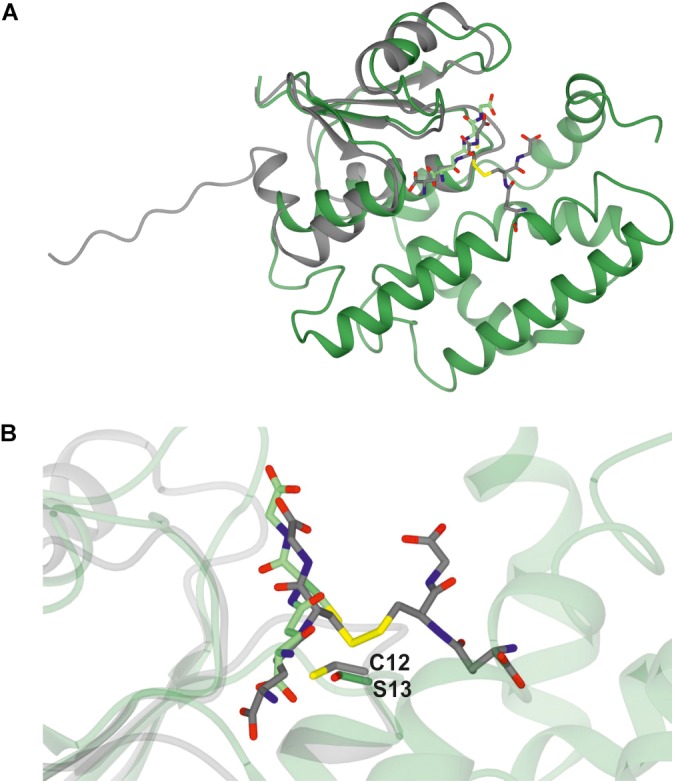
Comparison of GSTU25 with CoGRX2. **(A)** Superimposed structures of the glutaredoxin subunit from Clostridium oremlandii (CoGRX2 in complex with GSSG (C-atoms in gray), and the GSTU25 subunit (green) in complex with GSSG (C-atoms in green). The RMS value for the superimposed structures is 2.3 Å over 73 residues. **(B)** Position of the active residue for GSH thiol stabilization: serine 13, in GSTU25 and cysteine 12 in CoGRX2.

A *Clostridium oremlandii* glutaredoxin (*Co*GRX2) with two GSSG molecules per dimer has been reported (Lee et al., 2014) and exhibits significant similarity with GSTU25 at the core of the thioredoxin fold where four β-strands and α-helices, can be observed (Figure 3A). In GSTU25, a serine interacts with the GSSG molecule (Figure 3B), whereas in *Co*GRX2, cysteine acts as the GSH thiol stabilizer.

### Identification of Target Amino Acids for Site-Directed Mutagenesis

**FIGURE 4:**
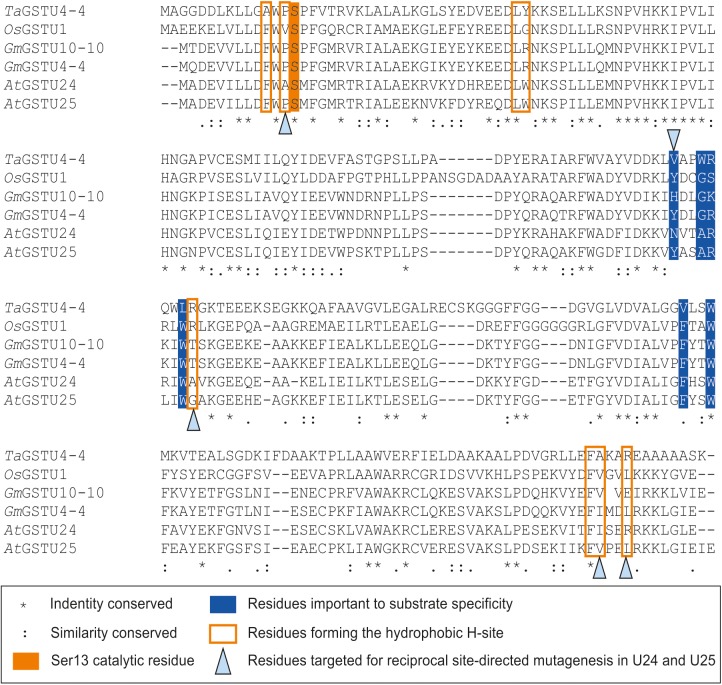
Multiple sequence alignment of Tau class GSTs. Figure generated using Clustal Omega (Sievers et al., 2011).

Seven key residues (F15, L37, K40, K53, E66, S67, R111) identified in the structure of GSTU25, and shown in Figure 2B with side-chain carbon atoms in blue, are all conserved in GSTU24. Comparisons with the structure of *Ta*GSTU4-4 (Thom et al., 2002) and *Gm*GSTU4-4 (Axarli et al., 2009) were used to highlight further amino acid residues in GSTU24 and GSTU25 that are likely to be involved in the formation of the hydrophobic H-site and thus in the determination of substrate specificity (shown as orange-outlined boxes in Figure 4). Of the six residues known to be important for substrate specificity in Tau class GSTs (shown in blue boxes), the residue at position 107 (N for GSTU24, Y for GSTU25) was the only one not identical between GSTU24 and GSTU25, and was thus targeted for mutagenesis. Subsequent homology modeling using the published structure of *Gm*GSTU4-4 (Axarli et al., 2009), which shares high (>60%) protein sequence identity with GSTU24 and GSTU25, identified four, further, non-identical residues, at positions 12, 115, 208 (209 for GSTU25) and 211 (212 for GSTU25), as shown in Figure 2B. The five amino acid residues targeted for reciprocal mutagenesis are listed in Table 3, marked as blue triangles in Figure 4, and highlighted with side-chain carbon atoms in gold for U25 in Figure 2B.

**Table 3:** Amino acid substitutions in the GSTU24 and GSTU25 mutants.

Enzyme	Mutation identifier	Substitution
GSTU24	A	A12P
	B	N107Y
	C	A115G
	D	I208V
	E	R211L
GSTU25	F	P12A
	G	Y107N
	H	G115A
	I	V209I
	J	L212R


### Activity of GSTU24 and GSTU25 Mutants Toward TNT

**FIGURE 5:**
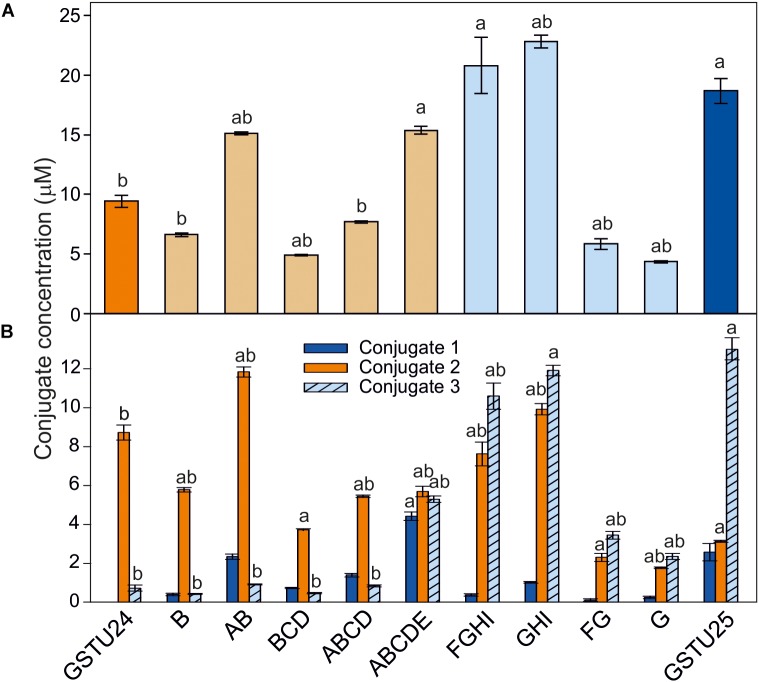
TNT-conjugate profiles from GSTs. **(A)** Total conjugates and **(B)** conjugate profiles produced by *At*GSTU24, *At*GSTU25, and mutants. Conjugate 3 = 2-glutathionyl-4,6-dinitrotoluene (GDNT). Results are means of three replicates ± SE, a, significantly different from *At*GSTU24, b, significantly different from *At*GSTU25.

To determine the effects of the target mutations on the ability of the GST proteins to produce the three different TNT-conjugates, the mutated proteins were assayed using TNT as substrate. For GSTU24, mutation BCD significantly reduced (*p* = 0.003) overall levels of conjugates produced to 52% of the wild-type GSTU24, whereas mutations AB and ABCDE displayed significantly higher (82 and 163%, respectively, *p* < 0.0001) conjugating activity than the wild type GSTU24 (Figure 5A). Figure 5B shows that all five mutants were able to produce conjugate 1, which was not detectable from wild type GSTU24 under these conditions. The mutant ABCDE was distinct from the others tested as it displayed the highest overall conjugating activity of the five U25-derived mutants. This ABCDE mutant was also able to produce significantly higher (*p* < 0.0001) amounts of conjugate 3, GDNT, than GSTU24, or the other four mutants. Moreover, ABCDE produced all three conjugates in almost equimolar concentrations.

For the GSTU25, mutations, FG and G significantly reduced (*p* < 0.0001) overall levels of conjugates produced to 31 and 24%, respectively, of the wild-type GSTU25. Compared to GSTU25, these FG and G mutants also yielded significantly reduced (*p* < 0.0001) overall levels of GDNT, while levels of conjugate 2 were not significantly affected for G (Figure 5B). Mutant GHI produced significantly more overall conjugates (*p* = 0.007) when compared to wild-type GSTU25, with both GHI and FGHI also producing significantly more conjugate 2 (*p* < 0.0001).

### Probing the GSTU24 and GSTU25 Mutants for Conformational Changes

**FIGURE 6:**
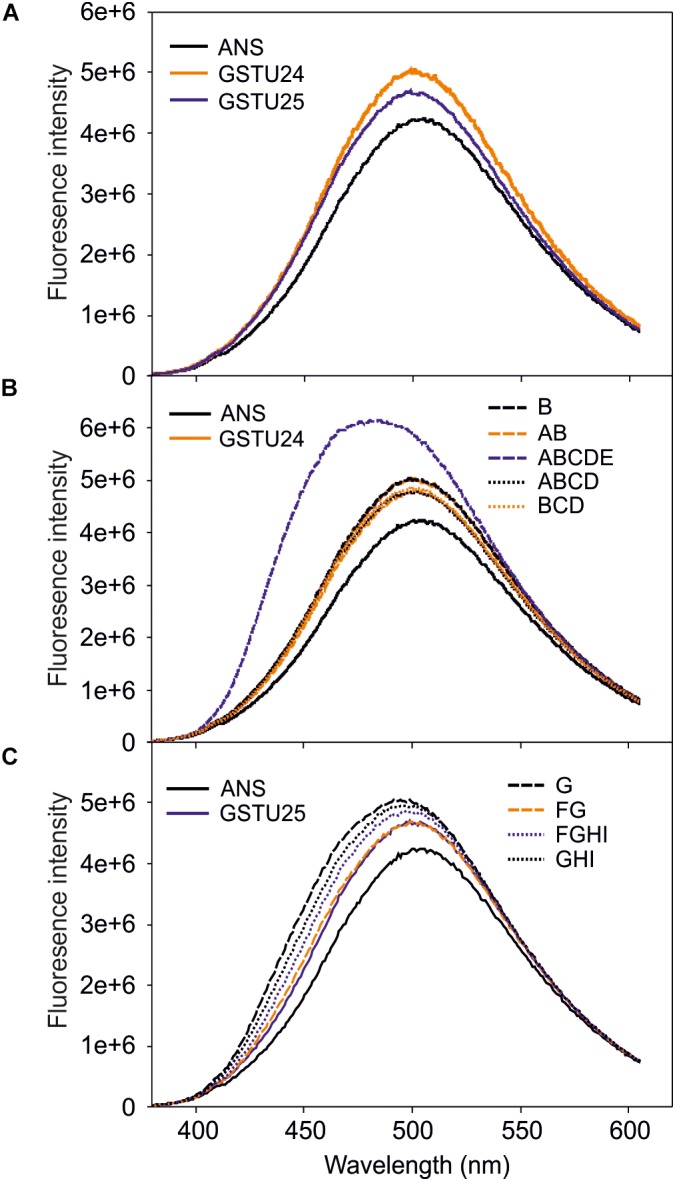
Fluorescence-emission spectra of 1-anilino-8-naphthalene-sulfonate (ANS) binding to the active site of the GSTs. **(A)** Spectra from GSTU24 and GSTU25. **(B)** Spectra from GSTU24 and its respective mutants. **(C)** Spectra from GSTU25 and its respective mutants. ANS, blank sample without enzyme; A-I, GSTU24 and GSTU25 mutants as presented in Table 3. Results are means of three technical replicates.

To identify any conformational changes in protein structure resulting from the presence of the mutated residues, the mutants were probed with 1-anilino-8-naphthalene-sulfonate (ANS) and the spectra measured. Both GSTU24 and GSTU25 shared a similar structure in the hydrophobic site (Figure 6A), with only the ABCDE mutant generating a significantly different fluorescence spectrum, indicative of a change in conformation (Figure 6B). The fluorescence spectra of the different GSTU25 mutants, varied slightly to one another, but none of them suggested a significant conformational change had occurred (Figure 6C).

### Activity of GSTU24, GSTU25, and Mutants Toward CDNB

**FIGURE 7:**
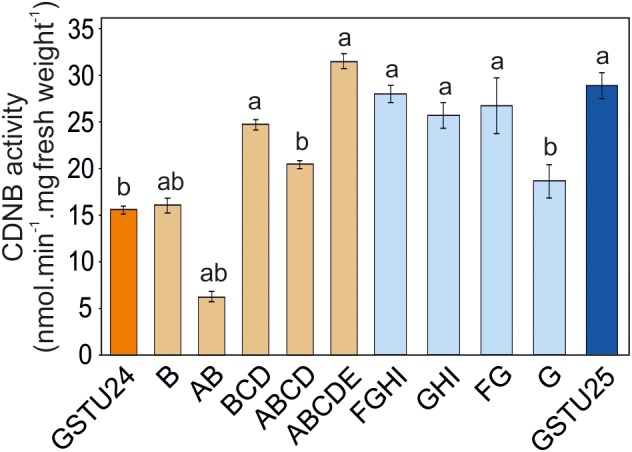
GST activity using 1 mM 1-chloro-2,4-dinitrobenzene (CDNB) substrate for GSTU24, GSTU25 and their respective mutants. Results are means of three technical replicates ± SE, a, significantly different from *At*GSTU24; b, significantly different from *At*GSTU25.

**FIGURE 8:**
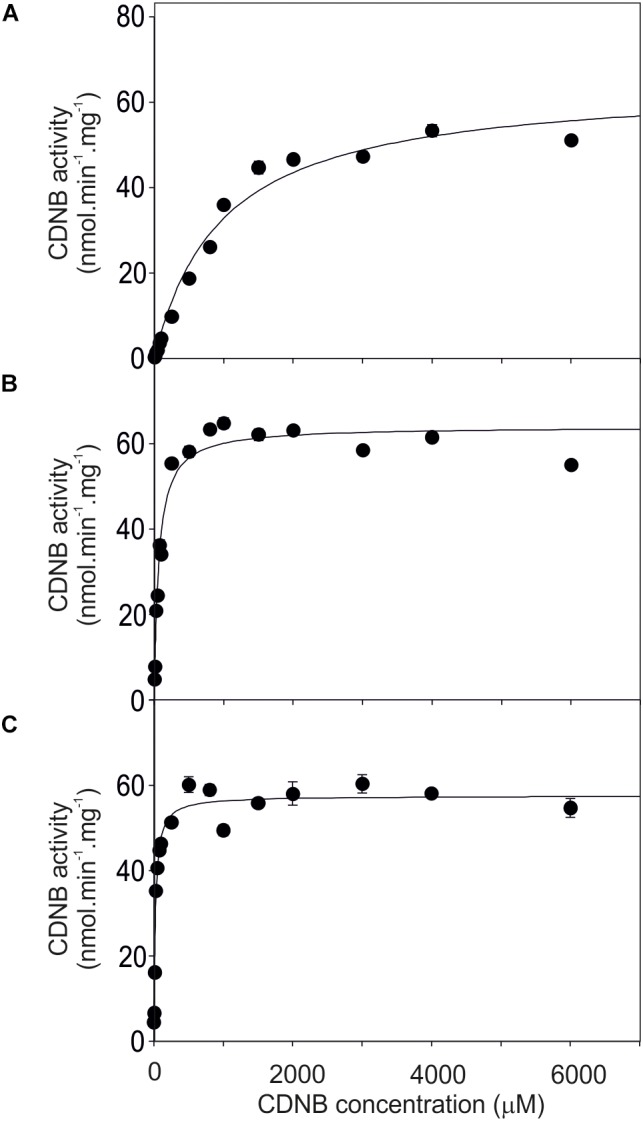
Michaelis–Menten plots from purified GST proteins. **(A)** GSTU24, **(B)** ABDCE mutant, and **(C)** GSTU25, assayed with 1-chloro-2,4- dinitrobenzene (CDNB) substrate. Values represent the mean of at least four reactions ± SE.

The activity of the mutants was measured using CDNB as a substrate. The results in Figure 7 show that all the mutants exhibited changes in activity that were significantly different to either, or both of the wild type GSTs. Given that the mutant ABCDE was distinct in displaying the highest overall conjugating activity, and producing significantly higher amounts of the desired target, GDNT, kinetic analysis was performed using CDNB substrate (Figure 8 and Table 4). While GSTU24 and GSTU25 exhibited similar V_max_ values, the *K*_m_ for GSTU24 was 45-fold higher than for GSTU25. In agreement with our reported conjugate profiles, the GSTU24 ABCDE mutant also displayed a reduced, GSTU25-like, *K*_m_ value.

**Table 4:** Enzyme kinetics for Figure 8, assayed using CDNB substrate.

Enzyme	*K*_m_ (μM)	V_max_ (nmole min^-^^1^ mg^-^^1^)	*R*^2^
GSTU24	972 ± 72.9	64.7 ± 1.6	0.98
ABCDE	64.8 ± 4.3	64.0 ± 0.8	0.96
GSTU25	21.5 ± 1.9	57.6 ± 0.7	0.95


## Discussion

The aim of this study was to identify the amino acids within GSTU25 involved in the formation of GDNT. To achieve this, the structure of GSTU25 was first determined. The structure, along with comparisons with the known Tau class GST structures *Ta*GSTU4-4 (Thom et al., 2002) and *Gm*GSTU4-4 (Axarli et al., 2009); and amino acid sequence of the closely related GSTU24, were used to highlight the amino acid residues in GSTU25 most likely to be involved.

### Crystal Structure of GSTU25

The electron density map for GSTU25 revealed unambiguously one GSSG per subunit. Within the GSTU25-GSSG structure, the GSH-1 moiety is stabilized by an arginine side chain (R111) while the GSH-2 moiety is located at a well-documented GSH binding site (Axarli et al., 2009, 2016; Skopelitou et al., 2015). The binding of GSTs to GSSG has been reported in the wood fungus, *Phanerochaete chrysosporium* *Pc*Ure2pA (Roret et al., 2015), and *E. coli* homologs *Ec*YghU and *Ec*YfcG. These bacterial and fungal GSTs have GSH transferase activity and are distantly related to glutaredoxins, redox enzymes that reduce disulfide bonds using glutathione (GSH) as an electron donor (Stourman et al., 2011). As shown in Figure 3, U25 shares significant similarity *Co*GRX2. In yeast (*Saccharomyces cerevisiae*), glutaredoxins *Sc*GRX1 and *Sc*GRX2 display GST-like activities, catalyzing the conjugation of CDNB to GSH (Collinson and Grant, 2003). As a multifunctional enzyme, exhibiting glutaredoxin, GPOX, and GST activities, GSTU25 would be well-suited to detoxify a wide range of the xenobiotics and oxidants present in diverse stress conditions.

### Residues Important to TNT-Conjugation Activity

Both GSTU24 and GSTU25 contain a serine residue (S13) in the active site at a position that allows it to stabilize the thiolate anion of glutathione. This is in agreement with structures of GSTs from Theta and Phi classes that are known to have GSH conjugating activity (Thom et al., 2001) and is replaced by cysteine for Lambda and DHAR GSTs (Dixon et al., 2002). The effects of the mutations on the activity toward TNT showed that Y107 in GSTU25 is important for conjugate specificity. GSTU24 does not produce conjugate 1 under the conditions tested; however, the N107Y mutation confers the ability to produce albeit small (6%) amounts of this conjugate. The data presented here also indicate that high activity of toward TNT requires both Y107 and P12. At the binding site of GSTU25, the GSSG subunits are located in a binding pocket surrounded by polar, non-polar and charged amino acids; a well-characterized active site for GSTs (Brock et al., 2013). In *Gm*GSTU4-4, the same S and Y residues of *Gm*GSTU4-4 are present in this binding pocket. When in complex with *S*-(*p*-nitrobenzyl)-glutathione, the S residue stabilizes the thiolate anion of GSH and enhances its nucleophilicity, while the Y residue is important in regulating catalytic function (Axarli et al., 2009). In GST25, L212 could also contribute to the production of GDNT; in *Gm*GSTU4-4, the close proximity of this residue to the nitro group of 4-nitrobenzyl (Axarli et al., 2009) could orientate TNT in the active site.

The five consecutive mutations present in GSTU25 ABCDE were predicted to engineer the near-complete active site of GSTU25 into GSTU24. The resulting conjugate profile and activity of ABCDE were similar to GSTU25 in that all three conjugates were produced, and at levels of overall conjugating activity similar to those of GSTU24. Furthermore, the *K*_m_ value of ABCDE was more in-line with that of GSTU25. Nonetheless, the fluorescence emission spectrum of ABCDE was significantly different from both GSTU24 and GSTU25, indicating a conformational change in the hydrophobic site, and TNT was not crystalized within the GSTU25 structure. Although TNT and reduced GSH were supplied during the crystallization process, incorporation of TNT into the active site was likely to have been hindered by the low aqueous solubility of TNT. Using synthesized, and more soluble, GDNT, in the absence of GSH, during the crystallization process could perhaps yield more information about the residues involved during the formation of this conjugate.

In summary, we have solved the structure for GSTU25, and identified key residues involved in the formation of 2-GDNT. Substitution of a nitro group for sulfur in 2-GDNT could render the aromatic ring more susceptible to subsequent degradation, and endogenous degradative pathways may already exist *in planta*. Alternatively, both bacteria and fungi are able to mineralize DNT (Serrano-González et al., 2018), and may have activity toward 2-GDNT. These fundamental studies will contribute toward the development of plant-based remediation strategies to degrade TNT, a toxic environmental pollutant.

## Author Contributions

KT, LA, and MD carried out the experiments. ER took the lead in writing the manuscript. All authors conceived and planned the experiments, provided critical feedback, helped shape the research, performed the analysis, and wrote the manuscript.

## Conflict of Interest Statement

The authors declare that the research was conducted in the absence of any commercial or financial relationships that could be construed as a potential conflict of interest.
